# SS18::SSX and BRD9 Modulate Synovial Sarcoma Differentiation

**DOI:** 10.3390/cells14242022

**Published:** 2025-12-18

**Authors:** Anna Kuntze, Victor Banerjee, Marcel Trautmann, Charlotte Pünt, Ruth Berthold, Pascal Hauser, Lucas Scholl, Eva Wardelmann, Kornelius Kerl, Wolfgang Hartmann, Ilka Isfort

**Affiliations:** 1 Gerhard-Domagk-Institute of Pathology, University Hospital Münster, 48149 Münster, Germany; 2 Department of Pediatric Hematology and Oncology, University Children’s Hospital Münster, 48149 Münster, Germany

**Keywords:** synovial sarcoma, SS18::SSX, BAF complex, GBAF, BRD9, E-Cadherin, Snail, Slug, differentiation, EMT

## Abstract

**Highlights:**

**What are the main findings?**

- SS18::SSX and BRD9 mediate E-Cadherin repression through regulation of Snail and Slug.
- SS18::SSX stabilizes Snail protein by preventing proteasomal degradation.
- BRD9 controls key pathways governing synovial sarcoma differentiation.

**What is the implication of the main finding?**

- Multilayered regulation of EMT factors by SS18::SSX and BRD9 contributes to the cellular phenotype in synovial sarcoma.

**Abstract:**

Synovial sarcoma (SySa) is a malignant soft tissue tumor that is characterized by an SS18::SSX fusion protein, which integrates into BAF chromatin remodeling complexes and alters global gene transcription. Despite its uniform genetic driver, SySa displays striking histomorphological and phenotypic heterogeneity, including spindle cell, glandular and poorly differentiated patterns. Prognosis is variable, with around 50% of patients developing metastases. Limited response to chemotherapy highlights the need for a better understanding of the underlying molecular mechanisms to guide alternative therapeutic strategies. Given the pivotal function of BAF complexes in SySa and their recently described impact on cellular differentiation processes, this study aims to investigate the role of SS18::SSX and specific BAF subunits in SySa differentiation. Nanostring analysis revealed that silencing of *SS18::SSX* and the GBAF subunit *BRD9* modulates the cellular differentiation pathways. SS18::SSX and BRD9 were found to regulate epithelial–mesenchymal-transition (EMT)-associated factors of Snail and Slug on different levels, with SS18::SSX repressing E-Cadherin expression. Published single-cell RNA sequencing data were analyzed to validate our finding that BRD9 contributes to SySa EMT regulation. Our study provides novel insights into the multilayered regulation of key EMT players by SS18::SSX and BRD9 in SySa, thereby defining tumor phenotype and (potentially) prognosis.

## 1. Introduction

Synovial sarcoma (SySa) is a malignant soft tissue tumor that predominantly affects young adults. Approximately half of all patients develop local recurrence or metastases [1,2,3]. Despite multimodal therapeutic approaches, prognosis for advanced disease remains poor, underscoring the need for targeted treatment strategies and a deeper understanding of the disease’s pathogenesis [4,5].

While otherwise genetically silent, the molecular hallmark of nearly all SySa is a characteristic reciprocal translocation, t(X;18)(p11;q11), between the *SS18* gene on chromosome 18 and a member of the *SSX* gene family on the X chromosome [6]. This rearrangement results in a SS18::SSX fusion protein that drives oncogenesis [7,8]. Histologically, SySa displays a broad morphological spectrum, including monophasic spindle cell tumors and biphasic neoplasms with epithelioid or glandular differentiation, while poorly differentiated round cell variants are rare (Supplementary Figure S1).

Over the years, several studies have investigated the cellular origin of SySa, proposing a mesenchymal lineage or a pluripotent origin. A mesenchymal lineage was suggested by Haldar et al., who demonstrated SySa development upon induction of SS18::SSX expression in myoblastic progenitor cells in a mouse model [9], and by Saito et al., who showed that SS18::SSX de-repression of E-Cadherin in 293T cells may represent a potential mechanism of mesenchymal-to-epithelial transition (MET) in SySa [10]. In contrast, a stem cell or pluripotent origin was proposed by Naka and colleagues, who reported that SS18::SSX globally dysregulates cellular differentiation, with SySa cell lines and human tumors expressing stem cell markers [11]. Accordingly, De Logu et al. suggested that SySa display a multipotent and partially neural differentiation [12].

Recently, substantial efforts have been made to identify epigenetic, genetic and transcriptional features associated with the distinct histologic phenotypes of SySa. Using bulk RNA and DNA sequencing, Chen et al. defined three molecular subtypes of SySa that differ in morphology and prognosis, largely reflecting the classical histological categories [13]: Subtype I—characterized by high mitotic activity, poor survival and a strong tendency to metastasize, corresponding to poorly differentiated tumors; Subtype II—predominantly monophasic, with a more favorable prognosis and comparably minor metastatic potential; and Subtype III—mainly biphasic, showing increased chemosensitivity, but associated with poor prognosis. Jerby-Arnon and colleagues performed the first published single-cell RNA sequencing analysis on 12 human SySa samples (obtained from 10 patients) and identified a “core oncogenic program” within mitotic cells that correlated with a poor clinical outcome [14]. Despite the availability of large-scale transcriptional datasets, the key molecular drivers and functional pathways underlying the morphological and prognostic heterogeneity of SySa remain largely unresolved.

BAF (mammalian SWI/SNF) chromatin-remodeling complexes, recognized as key regulators of development and cellular differentiation, are known to be frequently dysregulated or mutated across many cancer types [15,16]. The three major BAF complex subtypes share a common core structure centered around the ATPases BRG1 and BRM, with each complex incorporating distinct accessory subunits: GLTSCR1/GLTSCR1L and BRD9 are integrated in GBAF complexes; ARID1A/ARID1B and DPF2 in CBAF complexes; and PBRM1, ARID2 and BRD7 in PBAF complexes [17].

It is well established that the SS18::SSX protein replaces the wild-type SS18 protein, a subunit of the ATP-dependent BAF complexes GBAF and CBAF, while the PBAF complex lacks the SS18 subunit [18]. Incorporation of SS18::SSX into BAF complexes was found to promote the degradation of CBAF and to result in global dysregulation of chromatin accessibility and gene transcription [19,20]. Beyond this, recent studies revealed additional BAF-independent interactions between SS18::SSX and chromatin-associated proteins [21,22]. Among them, Tong et al. demonstrated that SSX1 binds to H2AK119ub-containing nucleosomes, thereby hijacking BAF complexes to Polycomb-associated chromatin regions [23].

Despite the advances in understanding chromatin dynamics, the mechanisms translating epigenetic perturbations into distinct morphological and clinical phenotypes remain elusive in SySa. Identifying targetable mediators of these processes, particularly those driving prognostically relevant phenotypes, is therefore of critical importance.

The present study focuses on an established EMT/MET regulatory axis involving E-Cadherin, Snail and Slug, which have previously been reported to be expressed in human SySa [24,25,26].

**Figure 1:**
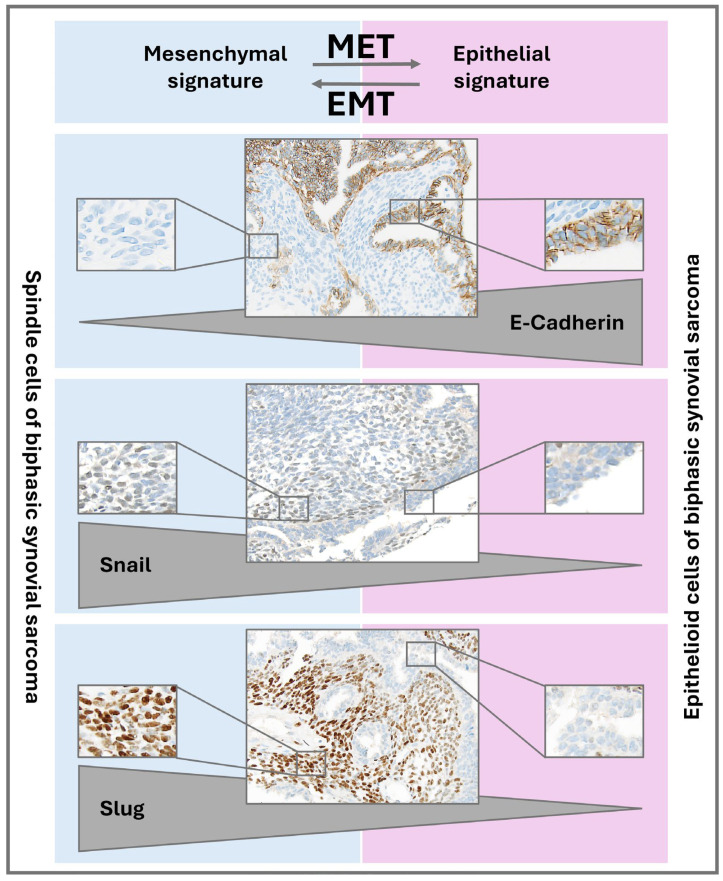
Expression of EMT markers E-Cadherin, Snail and Slug in epithelioid and spindle compartments of SySa. Schematic overview illustrating the differential expression of E-Cadherin, Snail and Slug in mesenchymal (**left**) versus epithelial (**right**) differentiation, indicated with arrowheads. Representative immunohistochemical stainings in biphasic synovial sarcoma are shown in the center, demonstrating E-Cadherin expression in epithelioid cells and Snail/Slug expression in spindle cells (original magnification: 1:200). Insets highlight representative spindle (**left**) and epithelioid (**right**) areas at higher magnification.

Figure 1 outlines their relevance for epithelial and mesenchymal differentiation and immunohistochemically shows their expression pattern in the spindle and epithelioid cells of SySa, respectively.

Given that BAF complexes constitute the central mediators of SS18::SSX-dependent oncogenesis and are considered to play a substantial role in cellular differentiation, their contribution to SySa differentiation will be investigated in the following section.

## 2. Materials and Methods

### 2.1. Cell Lines

Human SySa cell lines CME-1 (RRID: CVCL_N586, monophasic, SS18::SSX2), FUJI (RRID: CVCL_D880, monophasic, SS18::SSX2) and SYO-1 (RRID: CVCL_7146, biphasic, SS18::SSX2) were cultured as previously described [27] in Roswell Park Memorial Institute medium 1640 (RPMI 1640, supplemented with 10% fetal calf serum (FCS)) (CME-1 and FUJI) or in Dulbecco’s Modified Eagle Medium (DMEM, supplemented with 10% FCS) (SYO-1). HEK293T/17 cells (ATCC: CRL_11268, RRID: CVCL_1926) were cultured in DMEM (10% FCS). All cells were grown under standard conditions (37 °C and 5% CO2 in a humidified atmosphere) for a maximum of 25 passages. Testing for mycoplasma contamination was performed routinely via PCR. Cell line identity was verified by cell authentication single-nucleotide polymorphism profiling (a service performed by Multiplexion, Friedrichshafen, Germany) and, for SySa cells, also by regular detection of the specific SS18::SSX fusion via Western blot and RT-qPCR.

### 2.2. RNA Interference

Short interfering RNA (siRNA)-mediated knockdown of *SS18::SSX*, *BRD9*, *BRD7*, *PBRM1*, *ARID1A* and *ARID1B* was performed with combinations of prevalidated siRNAs, which were tested for their individual knockdown efficiency prior to pooling (individual knockdown efficiency in Western blot analysis is provided in Supplementary Figure S2). For si*SS18::SSX*, published duplex oligos were used (#1 = sense: 5′-CCAGAUCAUGCCCAAGAAGdTdT-3′; antisense: 5′-CUUCUUGGGCAUGAUCUGGdTdT-3′ [28], #2 = sense: 5′-UGACCAGAUCAUGCCCAAGdTdT-3; and antisense: 5′-CUUGGGCAUGAUCUGGUC AdTdT-3, [29]). Further siRNAs include *BRD9*#1 (Ambion™ Silencer™ Select siRNA, Thermo Fisher Scientific, Waltham, MA, USA), ID: s35293), *BRD9*#2 (Ambion, ID: s35294), *BRD9*#3 (Ambion, ID: s35295), *BRD7*#1 (Ambion, ID: s26524), *BRD7*#2 (Ambion, ID: s26525), *BRD7*#3 (s26526), *PBRM1*#1 (Invitrogen, Silencer™ Select; Thermo Fisher Scientific, Waltham, MA, USA, ID: s30400), *PBRM1*#2 (Invitrogen, ID: s30401), *PBRM1*#3 (Invitrogen, ID s30402), *ARID1A*#1 (Ambion, ID: s15785), *ARID1A*#2 (Ambion, ID: s15786) and *ARID1B* (Ambion, ID: s531279). Pooling of siRNAs was performed immediately prior to usage: si*SS18::SSX* (50% siRNA#1 and 50% siRNA#2), si*BRD9* (33% siRNA#1, 33% siRNA#2, 33% siRNA#3), si*BRD7* (33% siRNA#1, 33% siRNA#2, 33% siRNA#3), si*PBRM1* (33% siRNA#1, 33% siRNA#2, 33% siRNA#3) and si*ARID1A*+*ARID1B* (25% si*ARID1A*#1, 25% si*ARID1A*#2, 50% si*ARID1B*). Block-iT Alexa Fluor Red Fluorescent Control siRNA (Life Technologies, Thermo Fisher Scientific, Carlsbad, CA, USA, #14750100) served as a non-targeting negative control. Cells were grown in cell culture dishes overnight to reach a confluency of 30–40% and then transfected with pooled siRNAs for each target, respectively, using Lipofectamine RNAiMAX (Life Technologies), according to the manufacturer’s instructions. Culture medium was replaced 6 h after transfection to minimize cytotoxic effects of Lipofectamine. Subsequently, cells were incubated for 72 h post-transfection (standard; in the timeline experiment from 1 h up to 96 h) in their regular medium, supplemented with 2% FCS.

### 2.3. Compounds

MG132 (C26H41N3O5, Catalog No. S2619; Sellekchem, Houston, TX, USA) was dissolved in DMSO and used at a concentration of 5 µM for a time period of 6 h prior to cell lysis.

### 2.4. Transient SS18::SSX Fusion Gene Overexpression

Generation of the *SS18::SSX* expression plasmids was described previously [30]. HEK293T/17 cells were transfected with plasmid DNA (*SS18::SSX1* or *SS18::SSX2*) with the use of FUGENE 4K DNA transfection reagent, according to the manufacturer’s instructions. As a control, the appropriate amount of empty vector (EV) backbone (Life Technologies) was transfected. Cells were grown in 6 cm tissue culture plates for 48 h and the culture medium was changed immediately prior to transfection, from DMEM containing 10% FCS to DMEM containing 2% FCS. After transfection, the culture medium was replaced again after 3 h of incubation. Cells were incubated for 72 h post-transfection.

### 2.5. Cell Lysis and Immunoblotting

Cells were lysed with RIPA buffer (Cell Signaling Technology, Danvers, MA, USA) containing 1x Protease/Phosphatase-Inhibitor-Cocktail (Cell Signaling Technology, #5872) on ice for total protein isolation. The protein concentration was measured using a Bradford assay. Proteins were separated by SDS-Page and transferred to nitrocellulose membranes. After blocking with 5% milk powder in TBS-T, incubation with the following primary antibodies was performed overnight (4 °C): SS18::SSX (Cell Signaling Technology, #72364), BRD9 (Cell Signaling Technology, #58906), BRD7 (Cell Signaling Technology, #15125), PBRM1/BAF180 (Cell Signaling Technology, 91894), ARID1A (Cell Signaling Technology, #12354), ARID1B (Cell Signaling Technology, #92964), E-Cadherin (Cell Signaling Technology, #3195), Snail (Cell Signaling Technology, #3879), Slug (Cell Signaling Technology, #9585), GAPDH (Cell Signaling Technology, #5174) and β-actin (Sigma-Aldrich, St. Louis, MO, USA, #A5441). Incubation with horseradish peroxidase-linked secondary antibodies was performed at room temperature for 1 h. Signal detection was performed with the SignalFire ECL and Elite Reagent chemiluminescence detection kit (Cell Signaling Technology) and the Molecular Imager ChemiDoc system (Image Lab Software, version 5.1; Bio-Rad Laboratories, Hercules, CA, USA). All antibodies used in this study yielded specific bands at the molecular weights indicated by their respective manufacturers.

### 2.6. RNA Isolation, cDNA Synthesis and Quantitative Reverse Transcription PCR

Isolation of total RNA was performed with the RNeasy Plus Mini Kit (QIAGEN, Hilden, Germany, #74134), including DNase digestion. The RNA concentration was measured and the purity of the RNA was approved using UV spectrophotometry (NanoDrop, Thermo Fisher Scientific, Wilmington, DE, USA). Afterwards, cDNA synthesis with the ProtoScript II First Strand cDNA Synthesis Kit (New England Biolabs, E6560; Ipswich, MA, USA) was performed, according to the manufacturer’s instructions. The cDNA quality was approved with the detection of 28S ribosomal RNA on ethidium bromide gels. Quantitative reverse transcription PCR (RT-qPCR) was performed on a StepOnePlus Real-Time PCR System (Applied Biosystems, Applied Biosystems, Foster City, CA, USA) according to the manufacturer’s instructions, using the Power SYBR Green PCR Master Mix (Applied Biosystems, #4368708). Target gene expression was normalized to *HPRT1* as a reference gene (ΔCt). Statistical analysis between the two groups was performed on the ΔCt-values, using a paired two-tailed Student’s *t*-test on *n* = 3 biological replicates. Statistical differences were considered to be significant at *p*  <  0.05 (*), *p*  <  0.01 (**) and *p*  <  0.001 (***). ΔΔCt values and fold changes were used for graphical illustration only. RT-qPCR primer sequences are listed in Supplementary Table S1.

### 2.7. Nanostring Analysis

For Nanostring analysis, total RNA isolation was performed as described above with the RNeasy Plus Mini Kit (QIAGEN, #74134) including DNase digestion.

All materials for the analysis were obtained by Bruker (Billerica, MA, USA) for the nCounter Pro Analysis System. A predefined codeset with a special focus on pluripotency and differentiation pathways (nCounter XT CodeSet XT HS Stem Cell, Bruker, #115000499) was used, capturing 770 gene transcripts detected by fluorescent barcode-labeled hybridization probes. CodeSet hybridization and loading the Prep Station and the Digital Analyzer were performed according to the manufacturer’s instructions.

Normalization, differential gene expression (DE) and pathway analyses were performed with Nanostring nSolver™ 4.0 Analysis Software (Nanostring MAN-C0019-08), using the Nanostring Advanced Analysis Module 2.0 plugin (Nanostring MAN-10030-03), in accordance with the Nanostring Gene Expression Data Analysis Guidelines (Nanostring MAN-C0011-04). The Advanced Analysis Module 2.0 software is based on open-source R programs for quality validation, normalization, DE analysis, pathway scoring and gene-set enrichment analysis.

Reference genes for DE were selected with the geNorm algorithm, based on the global stability of pairwise expression ratios between samples. Dynamically 9–10 housekeeper genes with the best expression stability for each cell line, respectively, were used for normalization (Supplementary Table S2a) and normalized count data are provided in Supplementary Table S2b.

For DE, the gene-specific optimal statistical method (t-statistic) was calculated by the nSolver 4.0 differential expression data model (mixture negative binomial model, simplified negative binomial model or log-linear model). FDR *p*-value adjustment was performed with the Benjamini–Yekutieli method. The siRNA target (si*SS18::SSX*, si*BRD9*, si*BRD7*, si*PBRM1*, si*ARID1A+B*) was used as a categorical variable with the CTRL (non-targeting siRNA as a negative control, as described above) as the reference category. Analyses were tested with the replicate as a confounding variable, which was observed to have minimal effects on the DE results. Low count data (<20 counts) were removed before calculating DE and from further analyses.

Pathway scores represent the first principal component of the normalized expression of pathway genes, and gene-set analysis is a quantitative summary of pathway-specific DEs. The global significance score for each pathway was calculated as the square root of the mean squared t-statistic of genes directly via the nCounter advanced analysis 2.0 tool. The directed global significance scores reported here take the positive or negative value of the *t*-statistic into account, giving a tendency if a pathway is regulated into one direction.

Individual heatmaps were designed in GraphPad Prism (version 10; GraphPad Software, San Diego, CA, USA) with the use of directed global significance scores and DE data derived from the nCounter advanced analysis 2.0 tool.

### 2.8. Single-Cell RNA Sequencing

#### 2.8.1. Data Source

Single-cell RNA sequencing data were obtained from the Curated Cancer Cell Atlas (3CA) portal ([31], Weizmann Institute of Science). On 11 March 2025, we downloaded the dataset published by Jerby-Arnon et al. (2021) [14], comprising SySa samples profiled with both 10× Genomics (3 samples) and Smart-seq2 (12 samples), totaling 15 samples and 16,125 cells. Each dataset included three files: Exp_data_UMIcounts.mtx (10× Genomics) or Exp_data_TPM.mtx (Smart-seq2), Genes.txt and Cells.csv. Matrices were loaded, annotated with gene symbols and cell barcodes and converted into Seurat objects, using *CreateSeuratObject*. As all quality control and curation had already been performed by 3CA, we imported the processed objects directly without additional filtering. Metadata were incorporated and a technology label (10× Genomics or Smart-seq2) was assigned for downstream integration. The provided metadata did not include batch identifiers beyond sequencing technology. The full metadata table is provided in Supplementary Table S3a.

#### 2.8.2. Seurat Object Construction and Integration

All analyses were performed in R (≥4.3) [32]. The Seurat package (v5.1.0) [33] was used for dimensionality reduction, clustering and differential expression analysis. Study-specific Seurat objects (10× Genomics and Smart-seq2) were merged using the *Merge_Seurat_List()* utility (scCustomize v2.1.2) [34]. The initial integration involved standardizing the assays for each technology and identifying variable features within each dataset to ensure a balanced representation of shared biological structures. Anchors were computed using the reciprocal PCA (RPCA) [35] framework, which efficiently aligns cross-platform datasets by projecting each dataset into the PCA space of the other. These anchors captured shared transcriptional states between 10× Genomics and Smart-seq2 profiles while reducing platform-specific technical effects. To minimize potential technology-related biases and to prevent a disproportionate influence of the duplicated samples, further analyses were performed with a subset solely including the Smart-seq2 probes. Supplementary Figure S3 illustrates cluster-specific sample distribution and alignment for non-malignant clusters, while showing a sample-specific malignant cluster structure, as stated by Jerby-Arnon et al. [14].

#### 2.8.3. Cluster Assignment

Tumor clusters and non-tumor clusters were identified by gene expression signatures reported by Jerby-Arnon et al. [14] and tumor cells were further divided into groups (*Biphasic_glands*, *Biphasic_spindled*, *Monophasic*, *Poorly_differentiated* and *Mitotic_tumor_cells*) based on the expression of epithelial, mesenchymal and mitosis-related genes and on the designated tumor differentiation given in the publicly available metadata (Supplementary Table S3a).

#### 2.8.4. *AUCell* Analysis

Gene signatures representing the epithelial and mesenchymal programs were curated from Jerby-Arnon et al. (Supplementary Table S3b). *AUCell* was used to quantify epithelial and mesenchymal gene-set activity at single-cell resolution. Raw counts were ranked per cell, using *AUCell_buildRankings()*, and AUC scores for each gene set were computed with *AUCell_calcAUC()*. The resulting epithelial and mesenchymal AUC values were added to the Seurat metadata for downstream visualization. To visualize the relative transcriptional state, signature scores were min–max scaled to the interval [0, 1] and we calculated an Epithelial–Mesenchymal Ratio, defined as follows: Epithelial–Mesenchymal Ratio = Mesenchymal/(Epithelial + Mesenchymal + 10^−6^), resulting in values close to 0 for epithelial-like cells and close to 1 for mesenchymal-like cells. The scaled EMT ratio was displayed on the UMAP by using *ggplot* in a three-color gradient (epithelial → mixed → mesenchymal, Supplementary Figure S4a).

#### 2.8.5. Heatmap Analysis

To validate differentiation-related gene signatures between the identified subgroups, a heatmap was generated using *ComplexHeatmap*. Gene-wise expression values were z-score scaled, and hierarchical clustering was applied to both rows and columns. Rows were split according to predefined epithelial, mesenchymal and cell-cycle gene signature lists, as reported by Jerby-Arnon et al. (Supplementary Table S3b). Tumor cell clusters characterized by mitotic activity (referred to as *Mitotic_tumor_cells*, Supplementary Figure S4b) were excluded from subsequent differential gene expression analyses to avoid bias arising from their distinct transcriptional profiles.

#### 2.8.6. Differential Gene Expression Analysis

Differential gene expression analysis was performed between the four tumor subgroups (*Biphasic_glands*, *Biphasic_spindled*, *Monophasic* and *Poorly_differentiated*) after subsetting the data to exclude *Mitotic_tumor_cells* and *Non_tumor_cells*. Results were visualized using dot plots. For statistical analysis, gene expression levels between the predefined clusters were compared using the Wilcoxon rank-sum test. To account for multiple pairwise comparisons, the *p*-values were corrected using the Benjamini–Hochberg procedure. The adjusted *p*-values were used to assess the statistical significance. The statistical results are provided in Supplementary Table S4.

### 2.9. Immunohistochemistry

Immunohistochemical (IHC) staining was performed on 5 primary biphasic synovial sarcoma tissue specimens derived from the archive of the Gerhard Domagk Institute of Pathology (Münster University Hospital, Münster, Germany). Diagnoses were confirmed by SS18-break-apart FISH or RNA sequencing and all cases were reviewed by two experienced pathologists to confirm the differentiation status. The study was approved by the Ethics Review Board of the University of Münster (2023-528-f-S), and the experiments conformed to the principles set out in the World Medical Association Declaration of Helsinki and the United States Department of Health and Human Services Belmont Report.

For IHC, the automated Ventana BenchMark ULTRA IHC staining system (VENTANA/Roche) was used according to the manufacturer’s instructions, as reported previously [36]. In brief, 3-μm sections were deparaffinized and were pretreated with Cell Conditioning 1 solution (CC1, Ventana/Roche, Tucson, AZ, USA) for 24–64 min at 95–100 °C. Subsequently, incubation with primary antibodies was implemented for 16–32 min at 36 °C: E-Cadherin (Ventana, clone 36, no.05905290001, prediluted antibody, Tucson, AZ, USA), Snail (Cell Signaling Technology, #3879, predilution: 1:10) and Slug (Cell Signaling Technology, #9585, predilution: 1:100). Visualization of the immunoreaction was performed via the Optiview DAB IHC detection kit (ref. no. 760-700, Ventana/Roche). Counterstaining of tissue sections was performed with hematoxylin (ref. no. 790-2208, Ventana/Roche) and blueing solution (ref. no. 760-2037, Ventana/Roche).

## 3. Results

### 3.1. SS18::SSX and BRD9 Regulate EMT/MET Signaling in SySa Cells

**Figure 2:**
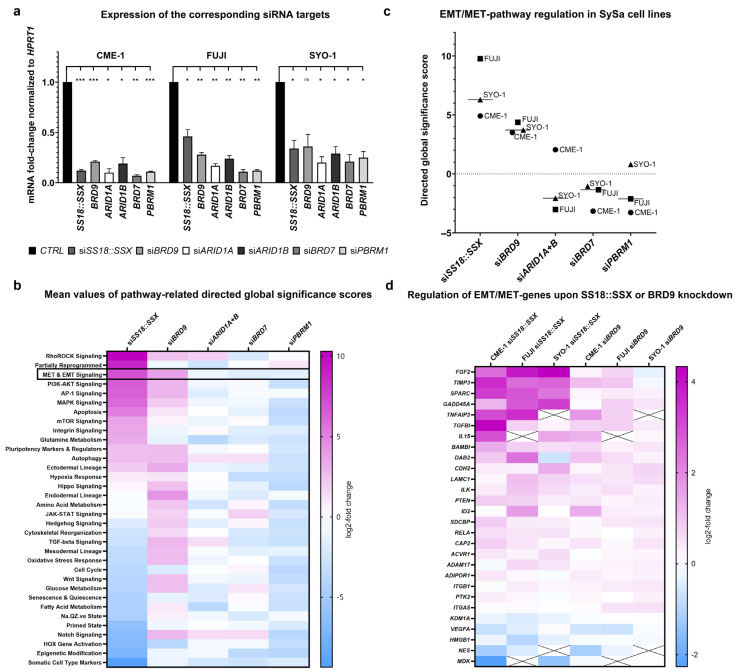
Effects of si*SS18::SSX* and silencing of BAF subunits on differentiation pathways. (**a**): Knockdown efficiencies upon silencing of *SS18::SSX* and specific BAF-subunits. mRNA expression (RT-qPCR) of the respective siRNA targets in SySa cell lines CME-1, FUJI and SYO-1 72 h after transfection with the indicated siRNAs: *SS18::SSX*, *BRD9* (GBAF), *ARID1A* (CBAF), *ARID1B* (CBAF), *BRD7* (PBAF) and *PBRM1* (PBAF), individually. Data are shown as mean fold change (2^−ΔΔCt^) + SEM of three biological replicates (*n* = 3). Statistical significance was determined by paired two-tailed *t*-test on ΔCt values (equal variance assumed); *p* ≥ 0.05 (ns = not significant), *p* < 0.05 (*), *p* < 0.01 (**) and *p* < 0.001 (***). (**b**): Regulation of different pathways upon silencing of *SS18::SSX* and specific BAF subunits. Table with mean values of directed global significance scores derived from CME-1, FUJI and SYO-1 cells upon knockdown of *SS18::SSX*, *BRD9* (GBAF), *ARID1A+B* (CBAF), *BRD7* (PBAF) and *PBRM1* (PBAF) (top), calculated by the Nanostring nCounter advanced analysis 2.0 tool. All pathways captured by the customer’s stem cell characterization panel are listed on the left. Colors are continuously scaled according to score-values. (**c**): si*SS18::SSX* and si*BRD9* increased EMT/MET pathway regulation. Scatter plot of individual directed global significance scores for CME-1, FUJI and SYO-1 upon knockdown of *SS18::SSX*, *BRD9* (GBAF), *ARID1A+B* (CBAF), *BRD7* (PBAF) and *PBRM1* (PBAF). Mean values for each siRNA are represented by horizontal lines. (**d**): Similar regulation of EMT/MET target genes upon *SS18::SSX* and *BRD9* knockdown. Heatmap of selected EMT/MET pathway-related genes with expression in at least two cell lines after knockdown of *SS18::SSX* and *BRD9,* respectively, in CME-1, FUJI and SYO-1 cells.

To evaluate the role of BAF complexes in SySa differentiation pathways, we performed a siRNA-mediated knockdown of specific BAF subunits, targeting all three complex subtypes: *BRD9* (GBAF), *ARID1A+ARID1B* (combined knockdown, CBAF), *PBRM1* (PBAF) and *BRD7* (PBAF). In addition, we targeted *SS18::SSX* using breakpoint-specific siRNAs in SySa cell lines. The knockdown efficiency in the SySa cell lines CME-1, FUJI and SYO-1 was demonstrated with different siRNAs (Supplementary Figure S2), with the highest efficiencies of combined siRNAs being 72 h post-transfection. All following experiments, unless otherwise specified, were conducted under the same conditions. The corresponding mRNA fold change values, presented in Figure 2a, confirm the high knockdown efficiencies.

We performed RNA-based Nanostring analysis of *SS18::SSX* and respective BAF subunit-knockdown samples using the manufacturer’s stem cell characterization panel. An overview of the directed global significance scores (mean values across all three SySa cell lines) is presented in Figure 2b; individual cell line data are provided in Supplementary Table S2c. As expected, silencing of *SS18::SSX* resulted in the most pronounced pathway alterations in all three cell lines. Among these, expression signatures of somatic cell-type markers (including *GATA4*, *GJA1*, *MAFA* and *MAP2*) and epigenetic modifications (comprising genes such as *CCNA2*, *CCNB1*, *DNMT1*, *DNMT3A/B*, *HDAC6*, *HDAC10* and *PHF2*) were the most downregulated upon SS18::SSX knockdown, whereas genes associated with RhoRock signaling (encompassing *HEG1*, *RHOC*, *THY1* and *ROCK1*), cellular reprogramming (referred to as “partially reprogrammed”, most regulated genes: *ATL1*, *CD44* and *COL3A1*) and EMT/MET signaling (including *FGF2*, *TIMP3*, *BAMB1*, *DAB2* and *COL1A1*) constituted the most upregulated pathways.

In comparison, knockdown of the different BAF complex type subunits resulted in less pronounced changes. However, all BAF subunits exhibited a varying degree of downregulation of epigenetic modifications upon knockdown, which is in line with their role as epigenetic regulators in SySa [37].

The depletion of specific subunits of CBAF (*ARID1A+ARID1B*) and PBAF (*PBRM1, BRD7*), respectively, predominantly resulted in a downregulation of gene clusters covered in this assay, with only a few signaling clusters being slightly upregulated. In contrast, the silencing of *BRD9* (GBAF complex) resulted in upregulation of several gene expression clusters—among them were RhoRhock signaling, EMT/MET signaling and AP-1 signaling.

Furthermore, the knockdown of *BRD9* induced the upregulation of differentiation pathways across the ectodermal, endodermal and mesodermal lineages, with highly consistent effects among the three cell lines (Supplementary Table S2c). In comparison, the silencing of *SS18::SSX* predominantly increased ectodermal lineage markers while decreasing mesodermal lineage pathways, though with some variability between cell lines. Overall, among the BAF subunits, *BRD9* depletion (GBAF) showed the greatest similarity to *SS18::SSX* knockdown, with both perturbations increasing EMT/MET signaling, which was a consistent finding across all SySa cell lines (Figure 2c).

A detailed analysis of EMT/MET pathway-related gene expression revealed regulatory similarities between *SS18::SSX* and *BRD9* knockdown. A heatmap with the most regulated EMT/MET pathway-related genes among all cell lines is shown in Figure 2d, including genes associated with epithelial differentiation (e.g., *TIMP3*, *TNFAIP3*, *BAMBI*, *CDH2*). *CDH1* (encoding E-Cadherin)—also included in the panel—was excluded from further differential gene expression analysis due to a low count number but exhibited a distinct increase in normalized count values with si*SS18::SSX* (CME: 255%, FUJI 207%, SYO-1 268%) and si*BRD9* (CME: 115%, FUJI: 180%, SYO-1: 108%). These data were calculated from the normalized count data (Supplementary Table S2b) and are additionally listed in Supplementary Table S2d.

### 3.2. Expressions of E-Cadherin, Snail and Slug Are Modulated by SS18::SSX and BRD9

**Figure 3:**
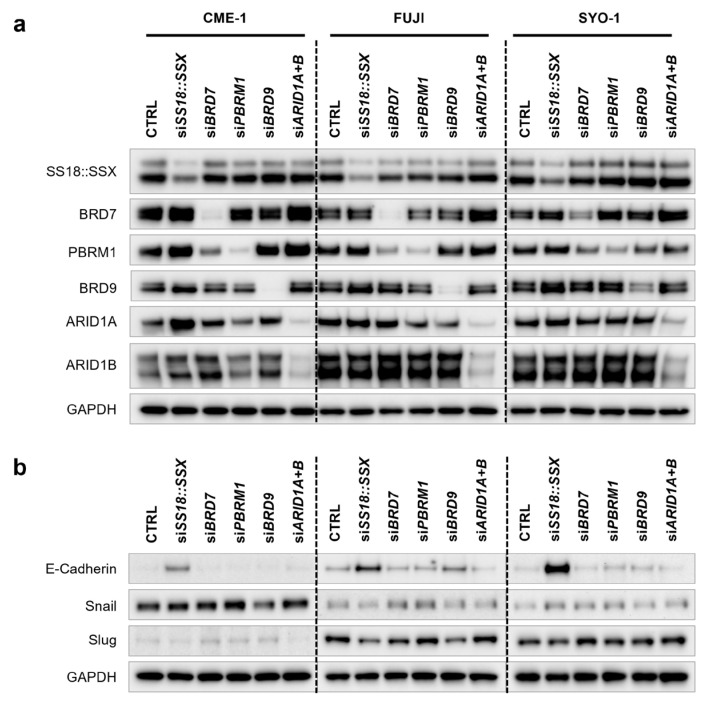
Knockdown of *SS18::SSX* and GBAF subunit *BRD9* modulates protein levels of E-Cadherin, Snail and Slug. (**a**): Protein levels of BAF subunits and fusion protein after transfection. Western blots showing decreased expression levels of target proteins 72 h after siRNA-mediated knockdown of *SS18::SSX*, *BRD9* (GBAF), *ARID1A+B* (CBAF), *BRD7* (PBAF) or *PBRM1* (PBAF), respectively, in three different SySa cell lines (CME-1, FUJI and SYO-1). A representative result out of three individual biological replicates is shown. (**b**): Protein levels of E-Cadherin, Snail and Slug after knockdown of *SS18::SSX* and specific BAF subunits. Western blots showing expression of E-Cadherin, Snail and Slug in CME-1, FUJI and SYO-1 cells 72 h after siRNA-mediated knockdown of *SS18::SSX*, *BRD9* (GBAF), *ARID1A+B* (CBAF), *BRD7* (PBAF) or *PBRM1* (PBAF), respectively. A representative result out of three individual biological replicates is shown.

To further investigate the role of BAF complexes in the regulation of EMT/MET signaling, we analyzed BAF- and SS18::SSX-dependent protein expression of the key epithelial–mesenchymal transition regulators E-Cadherin, Snail and Slug via Western blotting in SySa cell lines. Knockdown efficiencies on the protein level are shown in Figure 3a, demonstrating effective and specific protein downregulation 72 h post-transfection. Knockdown of individual BAF subunits had no consistent effect on the abundance of the other subunits despite *BRD7* siRNA, which reduced not only BRD7, but also PBRM1 protein levels, with both subunits being part of the PBAF complex.

Figure 3b shows increased E-Cadherin protein expression following *SS18::SSX* silencing in all three SySa cell lines. In the CME-1 and SYO-1 cell lines, basal E-Cadherin protein expression was very low and became clearly discernible upon the knockdown of *SS18::SSX*. Correspondingly, protein levels of the E-Cadherin repressors Snail and Slug were partly decreased in cells treated with *SS18::SSX* siRNA. Interestingly, knockdown of *BRD9* was also associated with a slight increase in E-Cadherin expression (FUJI) and reduced levels of Snail (CME-1) and Slug (FUJI). The knockdown of *BRD7*, *PBRM1* and *ARID1A+ARID1B* did not lead to consistent changes in E-Cadherin, Snail or Slug expression, confirming our Nanostring results.

**Figure 4:**
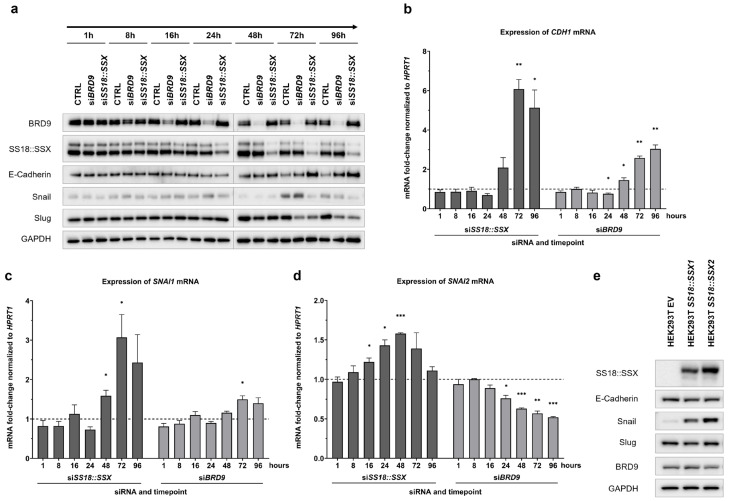
Regulatory effects of SS18::SSX and BRD9 on EMT targets depend on time point and knockdown efficiency. Induction of SS18::SSX protein increased abundance of Snail protein. (**a**): Knockdown efficiency and regulation of target proteins in the time-course in FUJI cells. Western blots showing time-dependent protein expression levels of SS18::SSX, BRD9 and proteins of interest upon silencing of *SS18::SSX* or *BRD9,* respectively. Temporal progression is illustrated by an arrow. A representative result out of three individual biological replicates is shown. (**b**–**d**): Time-dependent mRNA expression levels of *CDH1* (**b**), *SNAI1* (**c**) and *SNAI2* (**d**) upon silencing of *SS18::SSX* or *BRD9* in FUJI cells. Data are related to ΔCt values of control siRNA for each time point, respectively (RT-qPCR, fold change value for each control = 1, depicted as reference line). Statistical significance was determined by paired two-tailed *t*-test on ΔCt values (control siRNA vs. siBRD9 and control siRNA vs. siSS18::SSX, for each time point, respectively, equal variance assumed); data shown as mean ± SEM of fold change ((2^−ΔΔCt^)), *n* = 3. Only significant changes are labeled with *p* < 0.05 (*), *p* < 0.01 (**) or *p* < 0.001 (***). (**e**): Induction of SS18::SSX protein increased abundance of Snail protein. Western blot showing protein levels 72 h after plasmid transfection of *SS18::SSX1* or *SS18::SSX2* into HEK293T/17 cells. EV = empty vector transfection (control).

These findings are in contrast to a study by Saito et al. (2006) [10], who reported a decreased expression of *CDH1*, encoding E-Cadherin, upon depletion of *SS18::SSX*. To better understand and validate our findings, we performed a time-course analysis of both protein and mRNA levels, following *SS18::SSX* and *BRD9* silencing in FUJI cells. Samples were collected at 1, 8, 16, 24, 48, 72 and 96 h post-transfection (Figure 4a–d).

Significant downregulation of *SS18::SSX* and *BRD9* on the mRNA level was observed, starting from 8 h after siRNA transfection (Supplementary Figure S5), while protein level suppression was evident from 16 h (BRD9) and 24 h (SS18::SSX) onward (Figure 4a). Interestingly, *CDH1* mRNA levels showed a slight decrease at early time points, up to 24 h post-transfection (Figure 4b). However, after 48–96 h, when knockdown reached its maximal efficiency at protein level, *CDH1*/E-Cadherin expression markedly increased at both the mRNA and protein levels. This time-course analysis strengthens our observation that the silencing of *SS18::SSX* results in an induction of E-Cadherin expression. Simultaneously, the protein expression levels of Snail and Slug decreased upon *SS18::SSX* knockdown. As this effect was not mirrored by *SNAI1* (encoding Snail) and *SNAI2* (encoding Slug) mRNA expression (Figure 4c,d), a different mechanism of SS18::SSX might be involved to sustain Snail and Slug protein expression.

Induction of *CDH1*/E-Cadherin expression was also increased through *BRD9* knockdown at 48, 72 and 96 h post-siRNA transfection and was accompanied by decreased protein levels of Snail and/or Slug. Whilst the *SNAI1* mRNA expression was slightly increased, *BRD9* knockdown reduced the *SNAI2* mRNA levels. Thus, the decreased protein levels of Slug following si*BRD9* could be caused by reduced *SNAI2* gene expression, suggesting different regulatory mechanisms for *SNAI2* downstream due to *SS18::SSX* and *BRD9* depletion, respectively.

In summary, our results demonstrate that SS18::SSX and BRD9 both regulate E-Cadherin as well as Snail/Slug in SySa cells.

To further investigate our finding of SS18::SSX repressing E-Cadherin and enhancing Snail and Slug protein expression in a cell line with a completely different cellular background, we induced SS18::SSX1 and SS18::SSX2 protein expression in HEK293T/17 cells via transient plasmid transfection. *SS18::SSX1* or *SS18::SSX2* overexpression led to increased Snail protein levels compared to the control (Figure 4e). However, E-Cadherin protein abundance remained relatively stable 72 h after transfection, accompanied by a limited and non-significant increase in *CDH1* mRNA levels (Supplementary Figure S6). BRD9 protein expression remained constant throughout.

Taken together, cellular background plays a crucial role for SS18::SSX’s impact on E-Cadherin, while its effect on Snail accumulation appears to be cell-type independent.

### 3.3. SS18::SSX Stabilizes Snail by Preventing Its Proteosomal Degradation

Since knockdown of *SS18::SSX* results in decreased Snail and Slug protein levels while slightly increasing *SNAI1* and *SNAI2* mRNA expression in SySa cell lines, we hypothesized that SS18::SSX might stabilize Snail and Slug proteins by preventing their proteasomal degradation.

To test our hypothesis, we combined siRNA-mediated knockdown of *SS18::SSX* in SySa cell lines with a treatment of MG132 (carbobenzoxyl-L-leucyl-L-leucyl-leucinal), a potent 20S proteasome inhibitor [38]. As MG132 is known to reduce cell viability in various cell types [39]—an effect we also observed in SySa cells after 24 h—the compound was administered 6 h before sample collection, within the 72 h transfection interval.

**Figure 5:**
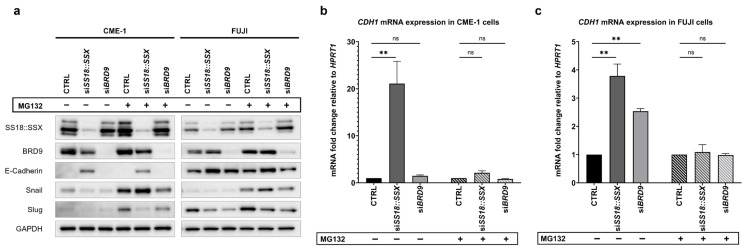
SS18::SSX stabilizes Snail protein to repress *CDH1* mRNA expression. (**a**): Proteasome inhibitor MG132 prevented decrease in Snail protein expression after knockdown of *SS18::SSX*. Western blots showing protein levels of targeted and EMT/MET-related proteins in CME-1 and FUJI cells 72 h after siRNA transfection with si*SS18::SSX*, si*BRD9* or control siRNA and 6 h after treatment with 5 µM MG132 (+) vs. DMSO (−). (**b**,**c**): MG132 administration diminished si*SS18::SSX*- and si*BRD9*-mediated increase in *CDH1*-mRNA expression. RT-qPCR results (fold change) of *CDH1* mRNA expression 72 h after siRNA transfection with si*SS18::SSX*, si*BRD9* or control siRNA (CTRL) and 6 h after treatment with 5 µM MG132 vs. DMSO (control) in CME-1 (**b**) and FUJI (**c**) cell lines. Data shown as mean ± SEM of fold change ((2^−ΔΔCt^)) of three biological replicates (*n* = 3). Reference values used for ΔΔCt are CTRL DMSO and CTRL MG132 (fold change = 1, respectively). Statistical significance was determined by a paired two-tailed *t*-test on ΔCt values (equal variance assumed); *p* ≥ 0.05 (ns = not significant) and *p* < 0.01 (**).

Western blot analysis (Figure 5a) revealed that Snail and Slug underly proteasomal degradation in SySa cell lines, as Snail and Slug protein levels increased under MG132 treatment. As observed in our previous experiments, knockdown of *SS18::SSX* reduced the Snail and Slug protein levels while E-Cadherin expression was induced in SySa cells in the DMSO control. Notably, the combination of *SS18::SSX* knockdown with the MG132 treatment led to Snail protein expression being preserved, which was associated with a reduction in *CDH1* mRNA levels in both cell lines (Figure 5b,c). Accordingly, protein levels of E-Cadherin showed a slight decrease in MG132-treated *SS18::SSX* knockdown cells compared to DMSO-treated si*SS18::SSX* cells (CME-1). In contrast, upon proteasome inhibition, Slug protein levels remained suppressed upon *SS18::SSX* knockdown, suggesting a different regulatory mechanism.

Our results indicate that SS18::SSX stabilizes the Snail and Slug protein levels which, in the case of Snail, is (at least partially) due to an inhibition of proteasomal degradation. As Snail and Slug are known repressors of E-Cadherin, this may lead to repressed E-Cadherin expression in SySa cells.

Eventually, the si*BRD9*-mediated reduction in Snail and Slug protein expression in CME-1 and FUJI cell lines could not be rescued by the application of MG132. This implies another regulatory mechanism of BRD9 on the Snail–Slug axis, which is obviously independent from mechanisms involving proteasomal degradation. Interestingly, in FUJI cells, MG132 diminished the increase in *CDH1* mRNA upon *BRD9* depletion.

### 3.4. Single-Cell RNA Sequencing Reveals Reduced Expression of BRD9 in Biphasic Tumors

**Figure 6:**
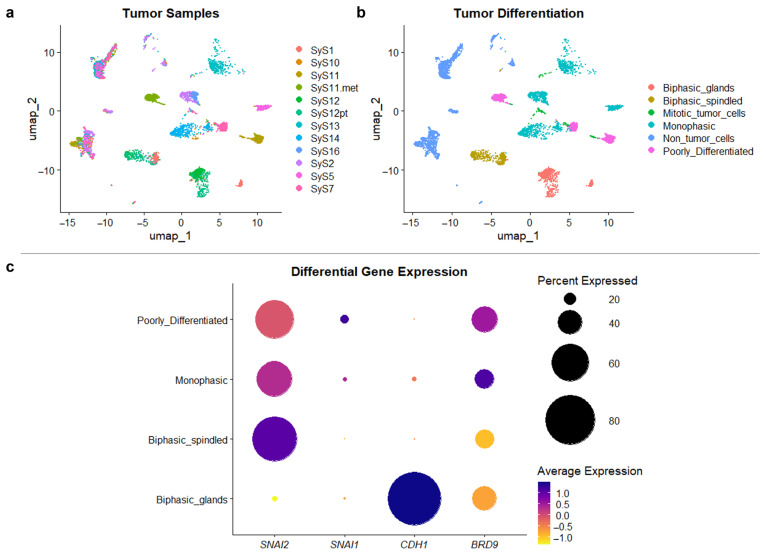
Differential gene expression, based on differentiation subtype of human SySa tumor cells. UMAP plots of single-cell RNA sequencing data (cohort of Jerby-Arnon et al.): (**a**): colored by tumor samples, (**b**): tumor differentiation (based on metadata), (**c**): dot plots showing differential gene expression of selected genes, stratified by cellular differentiation status.

Given the observed regulatory effects of BRD9 (GBAF) on EMT/MET pathways, we further investigated the potential correlations between BRD9 and cellular differentiation in human SySa tumor samples. To this end, we reanalyzed a publicly available single-cell dataset from Jerby-Arnon et al. [14], which includes 12 human SySa samples derived from 10 different patients. A UMAP of the tumor samples is shown in Figure 6a.

We applied the authors’ predefined histological subclassification (three biphasic tumors, seven monophasic tumors and two poorly differentiated tumors) to evaluate mRNA expression in relation to differentiation status. Gene expression-based analyses were used to identify epithelioid clusters within biphasic tumors and to validate the histological subclassification, revealing clear differences in transcriptional patterns among the *Biphasic_glands*, *Biphasic_spindled*, *Monophasic* and *Poorly_differentiated* subgroups (Supplementary Figure S4). In addition, non-tumor cells and mitotic tumor cells were identified and excluded from downstream analyses. Figure 6b shows the resulting UMAP, annotated according to the cellular differentiation state.

Dot plots demonstrate differential gene expression between the four tumor cell types (*Biphasic_glands*, *Biphasic_spindled*, *Monophasic* and *Poorly differentiated*), showing an inverse correlation between the expression of *CDH1* (high in *Biphasic_glands*) and *SNAI2* (low in *Biphasic_glands*) (Figure 6c). No significant expression of *CDH1* was observed in other tumor cell groups (*Biphasic_spindled*, *Monophasic* and *Poorly_differentiated*). *SNAI1* mRNA levels were generally low across all analyzed SySa samples, with the highest expression found in *Poorly_differentiated* tumors. In this dataset, the expression of *BRD9* was lower in biphasic tumor cells (*Biphasic_glands* and *Biphasic_spindled*) compared to monophasic and poorly differentiated cells. These results are in line with our finding of decreased *SNAI2*/Slug mRNA and protein expression upon *BRD9* silencing. However, since only two cases of biphasic SySa with clusters of epithelioid tumor cells and only two poorly differentiated tumors were included in the analysis, these observations must be interpreted with caution due to limited sample size.

## 4. Discussion

This study provides novel insights into the complex molecular mechanisms governing the differentiation processes in SySa and provides evidence for a regulatory role of the SS18::SSX fusion protein and BRD9, a specific subunit of GBAF complexes.

### 4.1. The Role of Different BAF Complexes in SySa Differentiation

The pivotal role of BAF complexes in the pathogenesis of SySa is well established. As major epigenetic regulators, they orchestrate stem cell function, differentiation and development through a tightly regulated interplay of their different subtypes—GBAF, CBAF and PBAF—governing global gene transcription and cell fate decisions [40].

We used a broad transcriptional analysis, performed on the Nanostring platform, to investigate the effects of SS18::SSX itself and the specific BAF complex subunits on cellular differentiation in SySa. Our analysis revealed the wide-ranging alterations of stem cell and differentiation pathways through the silencing of *SS18::SSX*, underscoring the capability of SS18::SSX to regulate cellular differentiation. BRD9 (GBAF) was shown to be the BAF subunit mirroring the most SS18::SSX-dependent pathway regulations; silencing resulted in the upregulation of differentiation pathways across the ectodermal, endodermal and mesodermal lineages. These results are in line with the known role of BRD9 in cellular development and the maintenance of stemness [41].

Moreover, we observed enhanced EMT/MET signaling following the knockdown of both *SS18::SSX* and *BRD9* (GBAF). Analysis of individual genes within the EMT/MET pathway showed that many genes were regulated in a similar fashion by both effectors, with increased expression of differentiation markers from the epithelial and mesodermal lineages. Our findings underscore the oncogenic role of BRD9 (GBAF) in SySa [20,42] and provide further evidence for (i) a multi-/pluripotent state of SySa with the capability to differentiate in a mesenchymal and epithelial direction and (ii) BRD9 (GBAF) to be a part of these regulatory processes.

### 4.2. SS18::SSX Mediates E-Cadherin Repression in SySa

In the following analyses, we focused on EMT/MET pathway regulators in SySa. Our experiments demonstrate a marked upregulation of E-Cadherin on the mRNA and protein level by silencing *SS18::SSX* across all analyzed SySa cell lines. Increased expression of E-Cadherin was associated with reduced protein levels of Snail and Slug, transcription factors that are highly expressed in various tissues and cancer types and that are well known to promote the epithelial-to-mesenchymal transition (EMT) and mesenchymal differentiation by inhibiting *CDH1* (E-Cadherin) expression [43,44].

Our findings contrast with those of Saito et al. (2006) [10], who reported that *CDH1* expression was decreased following *SS18::SSX* siRNA transfection in SySa cell lines; however, this effect was observed only 8 h post-transfection, representing a comparably early time point. In our time-course experiments, we also observed a slight reduction in *CDH1* expression at 8 h; however, this effect was not significant, particularly as the *SS18::SSX* knockdown itself was not yet detectable. The main effect that was attributable to *SS18::SSX* depletion became evident from 48 h post-transfection onwards, representing a clear induction of *CDH1*/E-Cadherin mRNA and protein expression, associated with a reduction in the Snail and Slug protein levels. Time-course experiments were performed with the SySa cell line FUJI displaying the highest level of E-Cadherin expression; however, comparable effects on E-Cadherin were found in the other SySa cell lines.

Moreover, Saito et al. [10] reported SS18::SSX-mediated induction of E-Cadherin via repression of Snail and Slug in HeLa cells, derived from an epithelial tumor [45], and in HEK293T cells, derived from an epithelial embryonic kidney cell line [46]. Given that these cell lines exhibit a differentiation background that is distinct from SySa cells, we likewise examined SS18::SSX induction in HEK293T cells.

Additionally, 72 h after plasmid transfection we were not able to observe significant enrichment in *CDH1*/E-Cadherin mRNA or protein levels. We propose that this may be due to the fundamentally distinct cellular context of differentiated epithelial cells (e.g., HEK293T cells), which lack the pluripotent and mesenchymal transcriptional signature characteristic of SySa cells. However, the SS18::SSX fusion protein highly induced Snail protein levels in HEK293T cells. This observation is in line with our previous findings and is evident despite the distinct cellular background of HEK293T cells.

Overall, we observed the si*SS18::SSX*-mediated expression of E-Cadherin in SySa, which was shown on the mRNA and protein level, accompanied by decreased Snail and Slug protein levels—major E-Cadherin repressors. Snail protein levels being elevated by SS18::SSX induction in non-SySa cells strengthens the evidence for a role of Snail in SySa pathogenesis acting through a mechanism independent from the cell context.

### 4.3. Regulation of Snail and Slug Protein Levels by SS18::SSX

The experiments conducted so far have shown that Snail and Slug can be regulated by the fusion protein. Upon knockdown of *SS18::SSX*, Snail and Slug protein levels decreased markedly. However, on the mRNA level, silencing *SS18::SSX* resulted in elevated *SNAI1* (Snail) and *SNAI2* (Slug) expression, indicating that SS18::SSX-mediated regulation likely occurs at a different level and that the observed increase in *SNAI1* and *SNAI2* transcripts may reflect a compensatory response. We therefore suggest additional (non-transcriptional) mechanisms for SS18::SSX to modulate Snail and Slug. Subsequent experiments revealed that the decrease in Snail levels mediated by the knockdown of *SS18::SSX* was abolished upon treatment with the proteasome inhibitor MG132. Thus, the fusion protein appears to stabilize Snail, while a distinct mechanism appears to underlie the Slug regulation, since the decrease in Slug levels is not prevented by MG132. Despite the short MG132 treatment of only 6 h, the si*SS18::SSX*-mediated increase in *CDH1* mRNA expression was completely diminished upon proteasome inhibition. In summary, our results suggest that SS18::SSX stabilizes Snail by preventing its proteasomal degradation as a mechanism of E-Cadherin repression. This stabilization might possibly be due to a direct interaction of SS18::SSX with Snail; such a protein interaction has been reported in HEK293T cells before [10]. Slug protein levels were reduced following *SS18::SSX* knockdown, even under proteasomal inhibition, indicating the involvement of a different regulatory mechanism that requires further investigation.

### 4.4. A Potential Role of BRD9 (GBAF) in Regulating EMT/MET in SySa

Since we provided evidence that BRD9 (GBAF) modulates SS18::SSX-dependent EMT/MET signaling in SySa, we aimed to analyze the effects of BRD9 in parallel with SS18::SSX. Knockdown of *BRD9* (GBAF) in SySa cell lines resulted in (i) reduced *SNAI2*/Slug mRNA and protein levels, (ii) decreased Snail protein expression and (iii) increased *CDH1*/E-Cadherin expression, indicating that GBAF contributes to EMT regulation in SySa, albeit with cell-line-specific differences. Our results are in line with the findings of Gatchalian et al., showing that BRD9-containing BAF complexes regulate the processes of cellular differentiation in mouse embryonic stem cells [47]. Moreover, Brien et al. discovered SySa to have a critical functional dependency on BRD9 by CRISPR-screen [42], whilst Michel et al. showed that the perturbation of BRD9 differs mechanistically from SS18::SSX disruption, postulating that GBAF complexes occupy fusion-independent sites across the SySa genome [20]. Since the reduced expression of Snail and Slug upon *BRD9* silencing could not be rescued by the proteasome inhibitor MG132—as observed for Snail upon *SS18::SSX* knockdown—we suggest that BRD9 acts through a distinct regulatory mechanism, potentially at the level of gene transcription. Further evidence will be required to determine whether the si*BRD9*-mediated decrease in Slug and Snail expression represents an SS18::SSX-independent mechanism in SySa.

It was recently reported that BRD9 is highly expressed in human SySa tissue samples [48]. Building on this observation, we provide additional evidence for a role for GBAF in SySa differentiation by re-analyzing single-cell RNA sequencing data from Jerby-Arnon et al.’s study [14]. We integrated the samples’ designated histological differentiation and epithelial/mesenchymal gene expression signatures to classify the tumor cells into four subgroups. Differential gene expression analyses revealed significantly lower mRNA expression of *BRD9* in spindle and epithelioid cells of biphasic tumors compared to monophasic and poorly differentiated tumors, respectively. As shown, expression of *CDH1* was clearly restricted to the epithelioid cells of biphasic tumors, while expression of *SNAI2* showed inverse distribution among the groups.

Taken together, we conclude that BRD9, similarly to the fusion protein, promotes increased levels of Snail and Slug while inhibiting E-Cadherin. Glandular cells of biphasic SySa demonstrated a decreased expression of *BRD9* (GBAF) associated with reduced *SNAI2* and high *CDH1* levels, further supporting our hypothesis of BRD9 being involved in SySa differentiation. These findings should be interpreted cautiously in the light of the limited sample size of the reanalyzed SySa cohort, including only two biphasic SySa. Therefore, a larger cohort of SySa tissue, including primary tumors representing all differentiation patterns, needs to be established to enable a more comprehensive assessment of the potential correlations between BAF complexes and differentiation signatures.

## 5. Conclusions

In summary, this study provides the first evidence that not only SS18::SSX but also BRD9, a specific subunit of GBAF complexes, play a role in the regulation of cellular differentiation in SySa. Our findings uncover a previously unrecognized mechanism of SS18::SSX-mediated E-Cadherin repression through the multilayered regulation of Snail and Slug. This mechanism may represent a key element in SySa oncogenesis and contribute to SySa’s unique transitional/multipotent cellular state at the interface between epithelial and mesenchymal differentiation.

## Data Availability

The publicly available scRNA sequencing data supporting the findings of this study were obtained from https://www.weizmann.ac.il/sites/3CA/sarcoma (accessed on 11 March 2025). Data are also available at DUOS (https://duos.broadinstitute.org/#/hom accessed on 11 March 2025) accession: DUOS-000123—as stated by the authors. The original contributions presented in this study are included in the article/Supplementary Materials. Inquiries can be directed to the corresponding author.

## Supplementary Materials

The following supporting information can be downloaded at: https://www.mdpi.com/article/10.3390/cells14242022/s1. Supplementary Figure S1. Representative histological sections of SySa. Hematoxilin and Eosin staining (original magnification: 1:200 and 1:400 as stated). Despite the same underlying fusion protein SS18::SSX, SySa occurs in distinct histomorphological phenotypes: monophasic (left), biphasic (center) and poorly differentiated/round cell variant (right) showing different growth patterns. At a higher magnification (1:400, bottom), cellular morphologies are visible with biphasic SySa, exhibiting spindle (#) and epithelioid (*) cells. Supplementary Figure S2. Protein levels of BAF subunits and the fusion protein upon transfection with individual siRNAs. Western blots showing decreased expression levels of target proteins 72 h after siRNA-mediated knockdown of *SS18::SSX*, *BRD9* (GBAF), *ARID1A* (CBAF), *ARID1B* (CBAF), *BRD7* (PBAF) or *PBRM1* (PBAF), respectively, in 3 different SySa cell lines (CME-1, FUJI and SYO-1). Supplementary Figure S3. UMAP plots and stacked bar plots of single-cell RNA sequencing data (cohort of Jerby-Arnon et al.): (a): UMAP plot colored by cluster. (b): UMAP plot colored by cell type annotation. (c,d): Stacked bar plots of cell types across Seurat clusters (c) and samples across Seurat clusters (d), illustrating how individual patient samples contribute to each cluster and cell type. Plots reveal alignment of non-tumor cells of different samples into specific clusters, whereas tumor cells exhibited separated clusters. Bars represent the absolute number of cells per type within each cluster. Supplementary Figure S4. Epithelial and mesenchymal gene signatures across differentiation groups. (a): UMAP plot showing scores of epithelial (purple) and mesenchymal (yellow) programs using *AUCell*. *AUC*-values for both signatures were min–max scaled to the range of 0–1, and a mix-ratio was calculated as Mesenchymal/(Epithelial + Mesenchymal). The resulting score ranges from 0 (epithelial-like) to 1 (mesenchymal-like). (b): Heatmap displaying z-score scaled expression of the top 30 differentially expressed genes per subtype (*Mitotic_tumor_cells*, *Biphasic_spindled*, *Monophasic*, *Poorly_Differentiated*, *Biphasic_glands* and *Non_tumor_cells*). Genes are grouped by malignant program (gene lists derived from Jerby-Arnon et al.: epithelial, mesenchymal, cell cycle and are listed in Supplementary Table S3b), as indicated by the row annotation color bar (purple = epithelial, yellow = mesenchymal, turquoise = cell cycle). Rows and columns are hierarchically clustered. Warmer colors represent higher relative expression (red), cooler colors represent lower expression (blue). Supplementary Figure S5. Time-dependent mRNA expression levels (RT-qPCR) of *SS18::SSX* (a) and *BRD9* (b) upon silencing of *SS18::SSX* or *BRD9*. Data shown as mean ± SEM of fold change (2^−ΔΔCt^) of three biological replicates (*n* = 3) and were related to ΔCt values of control siRNA for each time point, respectively (fold change value for each control = 1, depicted as reference line). Statistical significance was determined by paired two-tailed *t*-test on ΔCt values (control siRNA vs. siBRD9 and control siRNA vs. siSS18::SSX, for each time point, respectively, equal variance assumed, *n* = 3); *p* ≥ 0.05 (ns = not significant), *p* < 0.05 (*), *p* < 0.01 (**) and *p* < 0.001 (***). Supplementary Figure S6. *CDH1* mRNA expression levels (RT-qPCR) 72h after plasmid transfection of *SS18::SSX1* or *SS18::SSX2* into HEK293T/17 cells. Data shown as mean ± SEM of fold change (2^−ΔΔCt^) of three biological replicates (*n* = 3). EV = empty vector transfection (control). Statistical significance was determined by paired two-tailed *t*-test on ΔCt values (equal variance assumed); *p* ≥ 0.05 (ns = not significant), *p* < 0.05 (*), *p* < 0.01 (**) and *p* < 0.001 (***). Supplementary Table S1: Primer list. Supplementary Table S2: Nanostring data: a: selected housekeeper genes, b: normalized count data, c: directed global significance scores, d: *CDH1* normalized count data. Supplementary Table S3: Metadata provided by Jerby-Arnon et al. a: Sample specific data, b: gene signature lists. Supplementary Table S4: Single-cell RNA sequencing—statistics for differential gene expression by pairwise comparisons between tumor differentiation groups.

## Abbreviations

The following abbreviations are used in this manuscript:

SySa	Synovial Sarcoma
EMT/MET	Epithelial-to-Mesenchymal-Transition/Mesenchymal-to-Epithelial-Transition
FCS	Fetal Calf Serum
DMEM	Dulbecco’s Modified Eagle Medium
RPMI 1640	Roswell Park Memorial Institute Medium 1640
EV	Empty Vector
