# Supplementary figures and images for: SS18::SSX and BRD9 Modulate Synovial Sarcoma Differentiation
Source: Cells. 2025 Dec 18;14(24):2022. doi: 10.3390/cells14242022 (PMC12731587; doi:10.3390/cells14242022)

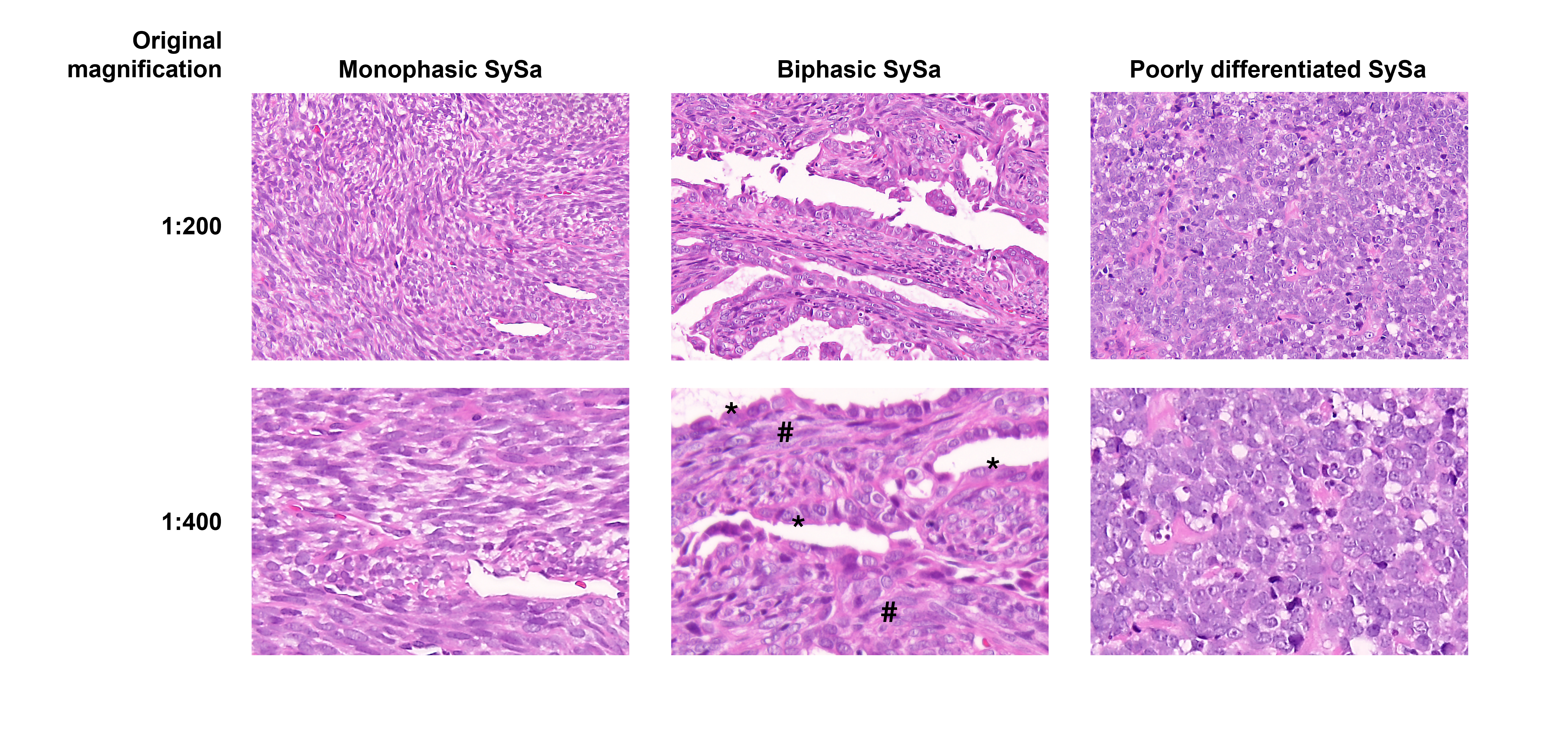

Supplement: Supplementary file 1 [file cells-14-02022-s001.zip › Figure_S1.png]

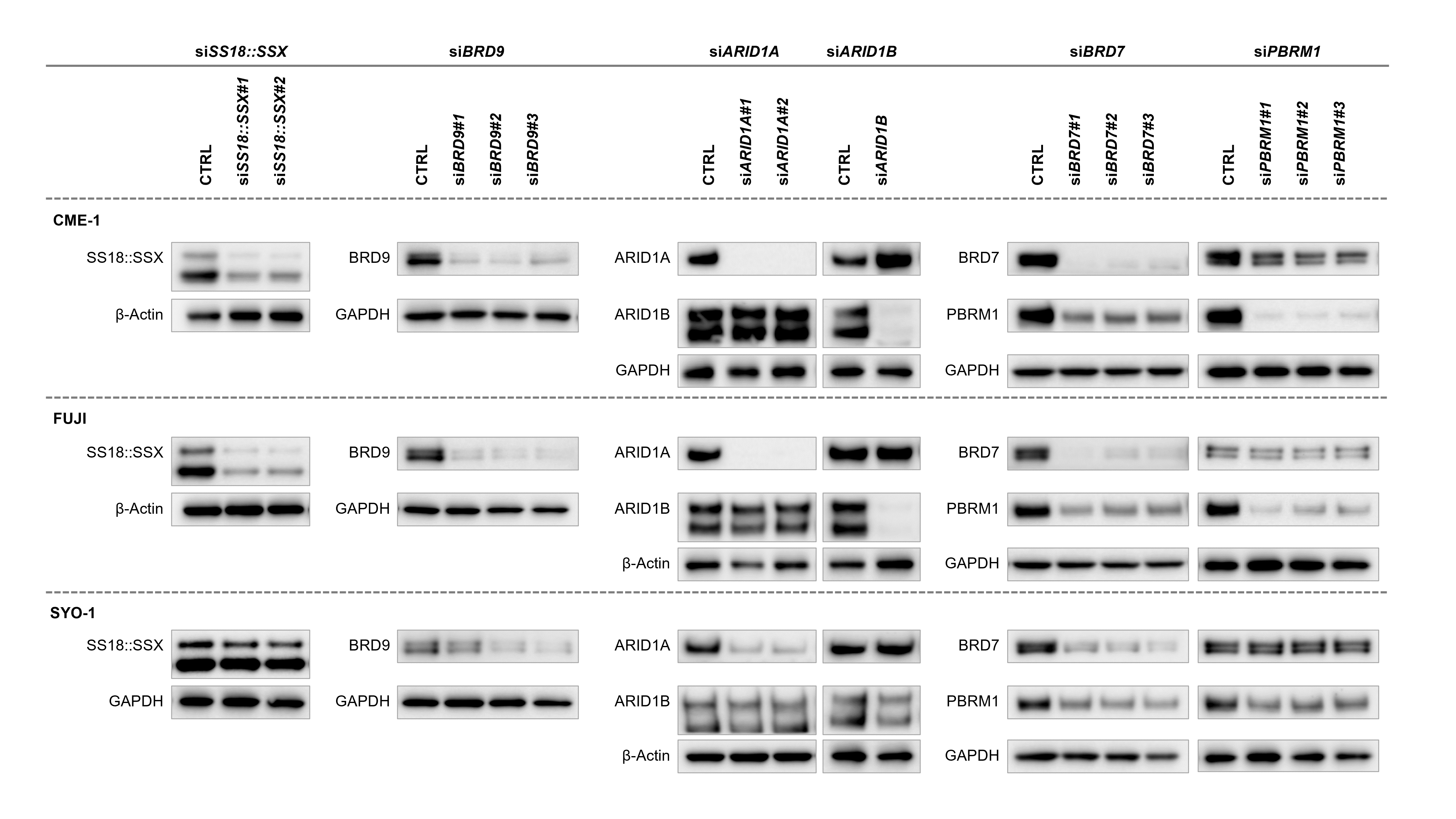

Supplement: Supplementary file 1 [file cells-14-02022-s001.zip › Figure_S2.png]

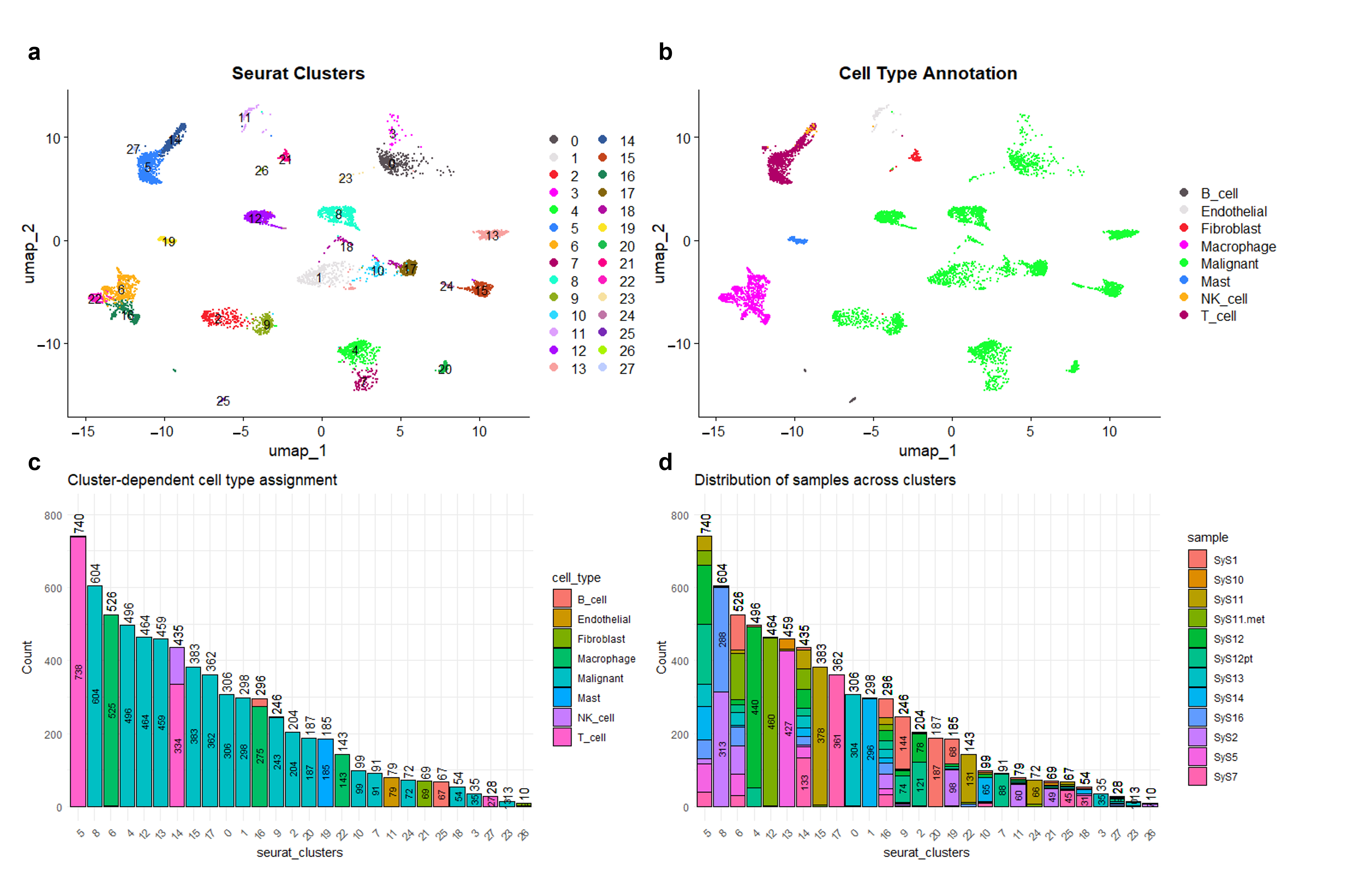

Supplement: Supplementary file 1 [file cells-14-02022-s001.zip › Figure_S3.png]

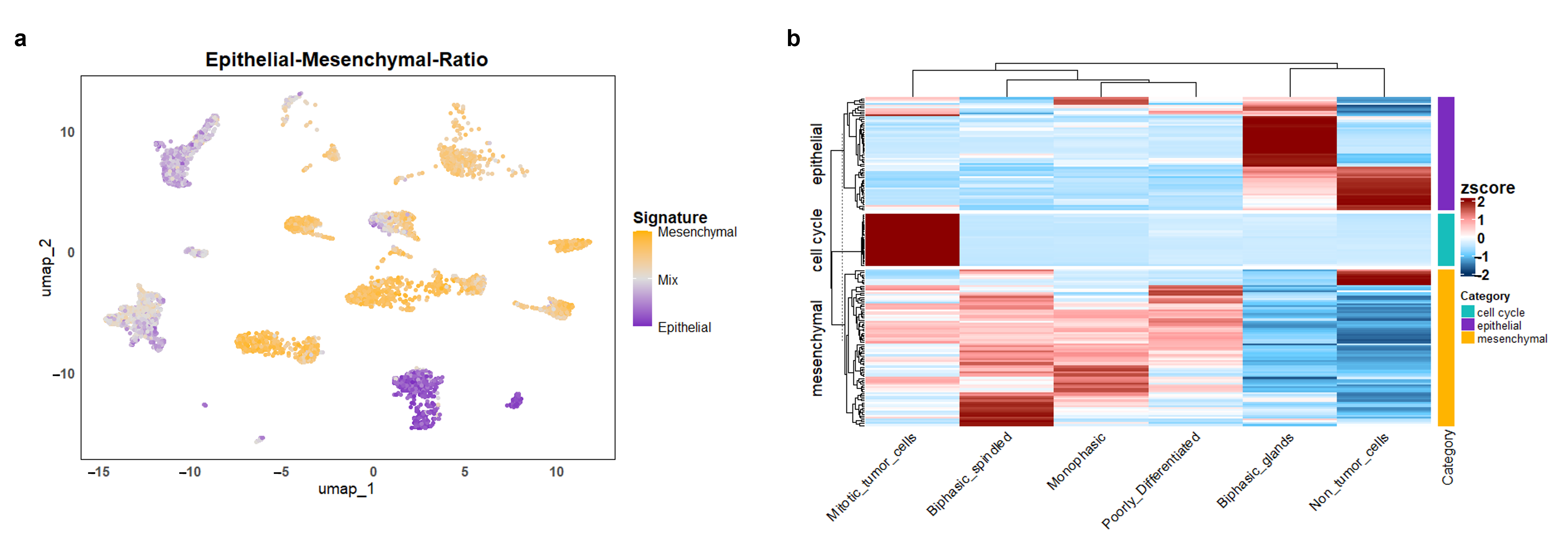

Supplement: Supplementary file 1 [file cells-14-02022-s001.zip › Figure_S4.png]

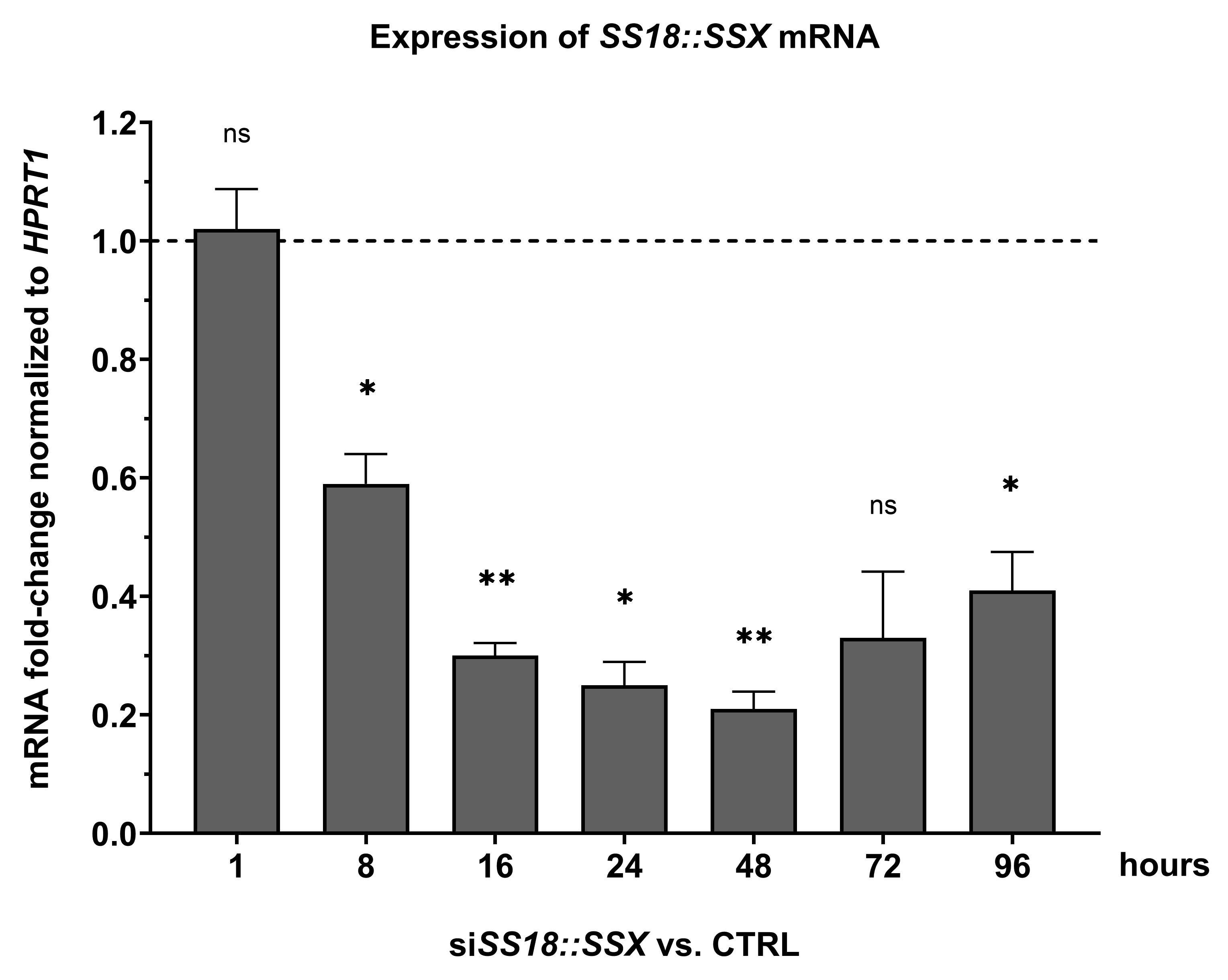

Supplement: Supplementary file 1 [file cells-14-02022-s001.zip › Figure_S5a.png]

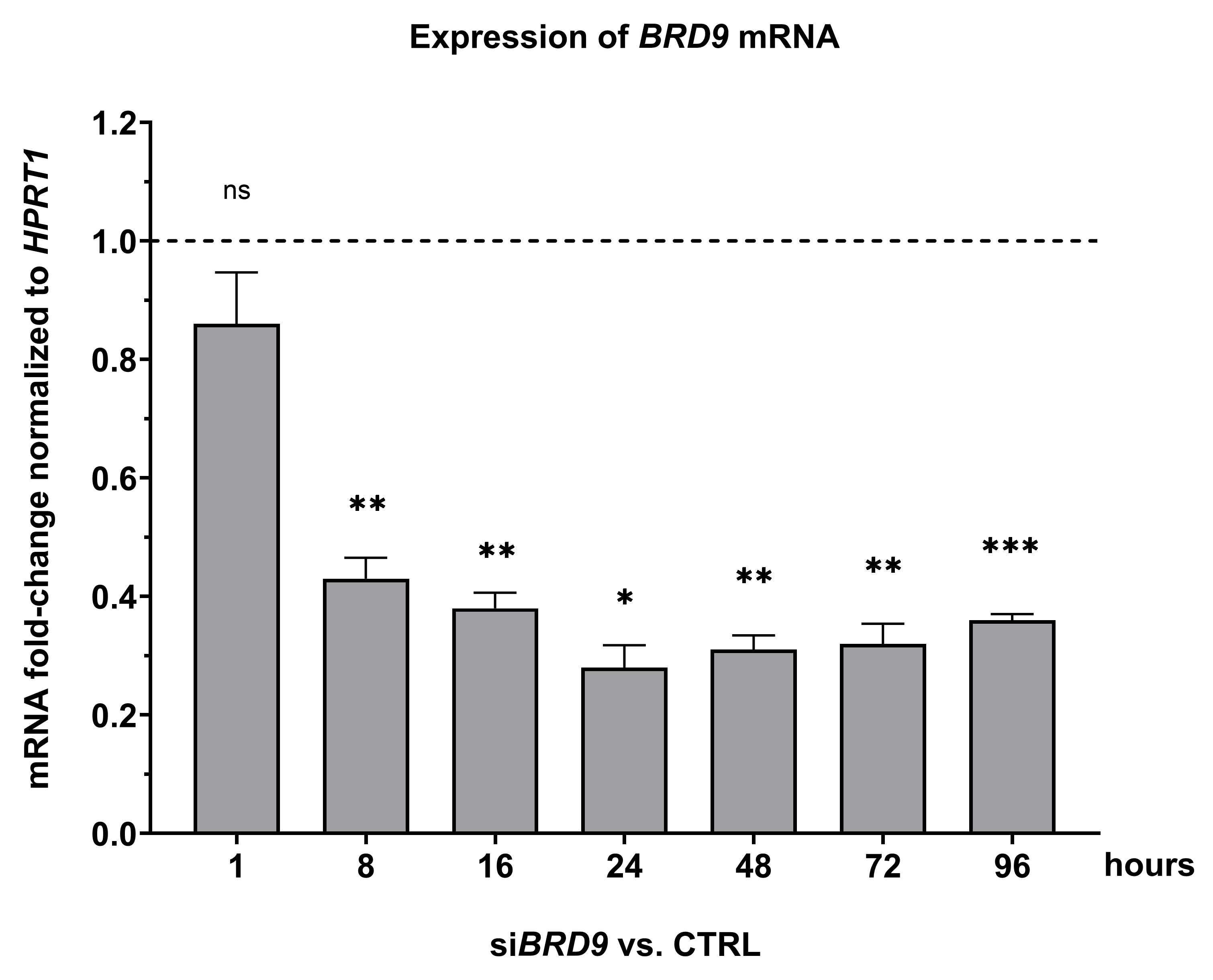

Supplement: Supplementary file 1 [file cells-14-02022-s001.zip › Figure_S5b.png]

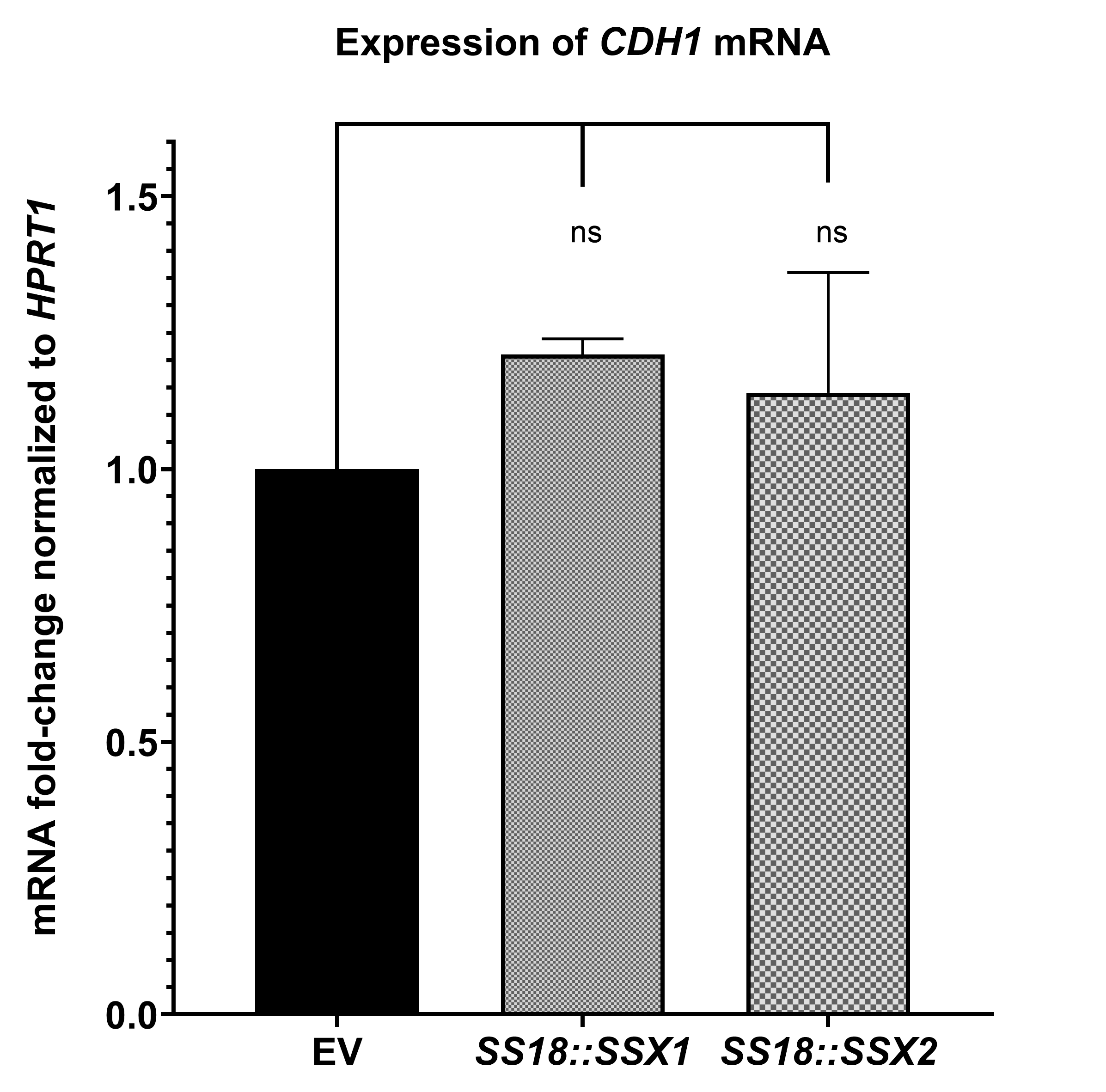

Supplement: Supplementary file 1 [file cells-14-02022-s001.zip › Figure_S6.png]
